# The complete chloroplast genome sequence of the mangrove associate species *Talipariti tiliaceum*

**DOI:** 10.1080/23802359.2021.1925171

**Published:** 2021-07-14

**Authors:** Zhimin Qiu, Yunxiao Zhu, Zhaokui Du, Pingyong Bao

**Affiliations:** a General Station for Forestry Technology Extension of Taizhou City, Taizhou, Zhejiang, China; b School of Life Sciences, Taizhou University, Taizhou, Zhejiang, China; c Zhejiang Provincial Key Laboratory of Plant Evolutionary Ecology and Conservation, Taizhou University, Taizhou, Zhejiang, China; d Green Eco-Environmental Science and Technology (Zhejiang) Co., Ltd., Ningbo, Zhejiang, China

**Keywords:** Chloroplast genome, mangrove associate species, phylogenetic analysis, *Talipariti tiliaceum*

## Abstract

*Talipariti tiliaceum* is an evergreen mangrove associate species distributed throughout the world. In this study, the complete chloroplast genome sequence of *T. tiliaceum* was assembled and characterized using high-throughput sequencing data. The chloroplast genome was found to be 161 766 bp in length, consisting of large single-copy (LSC) and small single-copy (SSC) regions of 89 273 and 19 551 bp, respectively, which were separated by a pair of 26 471 bp inverted repeat (IR) regions. The genome contained 129 genes, including 84 protein-coding genes, 37 tRNA genes, and 8 rRNA genes. A phylogenetic tree including 66 chloroplast genomes from various species revealed that *T. tiliaceum* was most related to *T. hamabo* of the same genus.

## Introduction

*Talipariti tiliaceum* (syn. *Hibiscus tiliaceus*), commonly known as the coast cotton tree or yellow mallow tree (Roychoudhury et al. 2017), is a typical mangrove associate species in tropical and subtropical coastal regions throughout the world (Abdul-Awal et al. 2016). The tree is fast-growing, salt- and wind-resistant, and is adapted to a wide range of mangrove environments, including inhospitable brackish swamps, waterlogged soils, and limestone-rich areas. *Talipariti tiliaceum* is commonly used as a traditional medicine for the treatment of fever, cough, abscesses, diarrhea, chest congestion, and ear infections (Hossain et al. 2015). Because of its medicinal properties, most previous research has focused on extraction methods, as well as the structural analysis and medicinal functions of its bioactive components in the leaves, flowers, and bark (Melecchi et al. 2002; Rosa et al. 2007; Li et al. 2008). Phylogeographical and genetic structural research based on the molecular biology of *T. tiliaceum* have also been reported (Tang et al. 2003; Takayama et al. 2006). However, there have been no reports on the chloroplast genome of *T. tiliaceum*, an important topic that would contribute to our understanding of the evolution of mangrove associate species.

Fresh young leaves of *T. tiliaceum* were collected from the National Nature Reserve for Mangroves in the Zhangjiang River Estuary (N23°55′, E117°26′), Yunxiao County, Fujian Province, China. Specimens were stored at the Herbarium of Taizhou University (Zhaokui Du, duzhaokui@qq.com) under the voucher number TZU-20200818HT01. The total genomic DNA was extracted from the leaves using a modified CTAB protocol as described by Doyle and Doyle (1987). After determination of its quality and concentration, the DNA was sheared into 400-600 bp fragments by ultrasonication. An Illumina paired-end library was constructed according to the manufacturer's instructions (Illumina, San Diego, CA, USA) and paired-end sequencing was performed using Hefei Biodata Biotechnologies Inc. (Hefei, China) on the Illumina HiSq platform. The raw reads were filtered using NGS QC Toolkit v2.2 software to obtain high-quality vector- and adaptor-free reads (Patel et al. 2012). The filtered chloroplast genome sequences were then assembled using the program NOVOPlasty 3.6 with the *T. hamabo* genome (GenBank accession no. NC_030195) as a reference (Dierckxsens et al. 2016). Annotation of the chloroplast genome was performed using DOGMA (Wyman et al. 2004) and NCBI-BLAST searches.

The complete chloroplast genome of *T. tiliaceum* comprised 161,766 bp of double-stranded, circular DNA (GenBank accession no. MN826059), containing two IR regions of 26,471 bp, separated by LSC and SSC regions of 89,273 and 19,551 bp, respectively. The overall GC content of the genome was 36.9% and it was predicted to contain 129 genes, including 84 protein-coding genes, 37 tRNA genes, and 8 rRNA genes. Six protein-coding genes (*ycf1*, *rps7*, *ndhB*, *ycf2*, *rpl2,* and *rpl23*), seven tRNA genes (*trnN-GUU*, *trnR-ACG*, *trnA-UGC*, *trnI-GAU*, *trnV-GAC*, *trnL-CAA,* and *trnI-CAU*), and four rRNA genes (*rrn5*, *rrn4*, *rrn23,* and *rrn16*) were duplicated in the IR regions. Nine genes contained two exons and four genes contained three exons.

**Figure 1.**
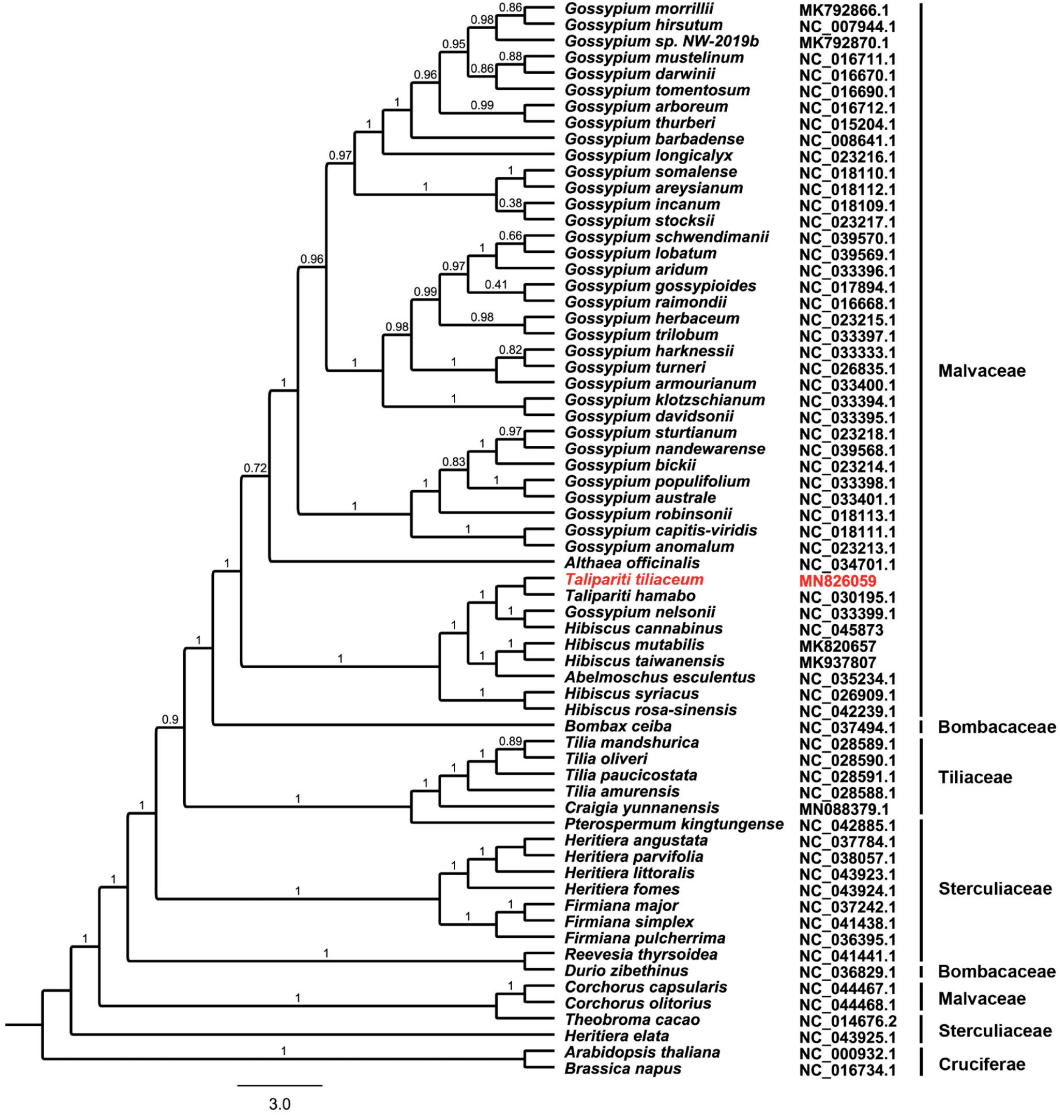
Phylogenetic tree inferred by the maximum likelihood (ML) method based on 89 single-copy genes from 66 representative species. *A. thaliana* and *B. napus* were used as out-groups. A total of 1000 bootstrap replicates were computed; the bootstrap support values are shown at the branches.

A total of 65 complete chloroplast genomes were downloaded from GenBank to investigate the phylogenetic relationships of *T. tiliaceum* within the genus *Talipariti* and other related groups. All the chloroplast genome sequences were aligned using MAFFT v7.307 (Katoh and Standley 2013). A maximum-likelihood (ML) phylogenetic tree was constructed based on a dataset of single-copy genes that included 62 coding sequences, 25 tRNAs, and 2 rRNAs by FastTree version 2.1.10 (Price et al. 2010). *Arabidopsis thaliana* and *Brassica napus* were used as out-groups. The phylogenetic analysis showed that *T. tiliaceum* was most closely related to *T. hamabo* (Figure 1). The sequencing of the chloroplast genome of *T. tiliaceum* may contribute to a better understanding of the evolution of *Talipariti* species within Malvaceae family.

## Data Availability

The genome sequence data that support the findings of this study are openly available in GenBank of NCBI at (https://www.ncbi.nlm.nih.gov/) under the accession no. MN826059. The associated BioProject, SRA, and Bio-Sample numbers are PRJNA698892, SRR13610058, and SAMN17761689, respectively.
